# Prognostic Immune Cell Profiling of Malignant Pleural Effusion Patients by Computerized Immunohistochemical and Transcriptional Analysis

**DOI:** 10.3390/cancers11121953

**Published:** 2019-12-05

**Authors:** Chengguang Wu, Fabian Mairinger, Ruben Casanova, Aashil A. Batavia, Anne-Laure Leblond, Alex Soltermann

**Affiliations:** 1 Institute of Pathology and Molecular Pathology, University Hospital Zurich, CH-8091 Zurich, Switzerland; ChengGuang.Wu@usz.ch (C.W.); ruben.casanova@uzh.ch (R.C.); Aashil.Batavia@usz.ch (A.A.B.); Anne-Laure.Leblond@usz.ch (A.-L.L.); 2 Institute of Pathology, University Hospital Essen, D-45147 Essen, Germany; Fabian.Mairinger@uk-essen.de

**Keywords:** malignant pleural effusions, cytopathology, immune profiling, digitalized image analysis, prognosis, immunomodulator

## Abstract

Malignant pleural effusion (MPE) is a severe condition of advanced tumors without effective therapy. We used digitalized immunohistochemical and transcriptional approaches to investigate the prognostic influence of immune cells and expression variance of associated immunomodulatory molecules in MPE. Cytology tissue microarrays were constructed from MPE cell blocks of 155 patients with five tumor entities. Immune cells lineage markers were quantified by computational cytopathology on immunohistochemistry. mRNA expression analysis of nine lineage markers and 17 immunomodulators was performed by NanoString. Immunohistochemically quantified high B cells to leukocytes ratio (hazard ratio (HR) = 0.70, *p*-value = 0.043) and low neutrophils to leukocytes ratio (HR = 1.78, *p*-value = 0.003) were favorable prognosticators for overall survival independent of tumor entity. Correspondingly, patients with high B cells but low neutrophils gene expression signature showed longer median overall survival of 500 days (HR = 2.29, *p*-value = 0.009). Regarding targetable molecule expressions, lung adenocarcinomas were characterized by high PD-L1, but mesothelioma by high *LAG-3*. Ovarian carcinoma was least immunogenic. Independent of tumor entity, the condition of the immune system in MPE liquids is able to provide additional prognostic cytologic information. Combined analysis of lineage specific markers and related immunomodulators may direct immune-based therapeutic decisions.

## 1. Introduction

Patients bearing malignant pleural effusion (MPE) have a dismal overall survival (OS) with median values ranging from 3–12 months [1] since tumor spread to the pleural cavity is defined as a late stage IV disease. However, individual survival times vary depending on the tumor entity. It has been shown that from the onset of effusion lung adenocarcinoma MPE patients had a worse OS compared to gynecologic carcinoma and mesothelioma; gynecologic carcinoma OS was worse than mesothelioma [2].

Etiologically, MPEs most frequently result from the pleural spread of carcinomas from lung, breast, ovary, and stomach origin. Mesothelioma is the main primary cancer [3]. Next to systemic therapy, intra-cavitary combined drug administration (e.g., cisplatin loaded to a fibrin carrier), has been evaluated and shows reduced systemic adverse effects while local cytotoxic concentrations are maintained [4,5,6]. Since MPEs are confined liquid environments containing the full spectrum of immune cells, localized immune therapy could be envisaged as a novel treatment option.

Regarding immunity, few studies have investigated the MPE immune cells composition of the different tumor entities. A high prevalence of functionally suppressive CD4+ CD25+ T-cells was observed in lung and breast carcinoma but not in mesothelioma MPEs, which in turn contained more activated CD8+ T-cells and increased expression levels of TGF-beta [7]. Moreover, the roles of other cytokines such as IL-17 and soluble B7-H3 in MPE have recently been emphasized in terms of their diagnostic and prognostic potentials [8,9]. Therefore, detailed knowledge of individual MPE immune cells composition is required for tailored immune therapy in order to overcome immunosuppressive mechanisms.

Immunomodulatory molecules play key roles in cancer-inhibiting or cancer-promoting responses. Thus, the evaluation of immune checkpoints as well as the expression of immune stimulators and their interconnections in each MPE tumor type is desired. For instance, the immunosuppressive function of the immune checkpoint PD-1/PD-L1 pathway has been demonstrated in lung cancer pleural effusions, by promoting dysfunction of tumor-responsive T-cells [10]. However, other relevant immune therapeutic targets such as CTLA-4, OX40, LAG3, and CD40 remain to be investigated. Therefore, profiling of immune checkpoints and stimulators in MPE may increase the success rate of local or even systemic immune therapy.

The aim of our study was to perform a comprehensive immune cell and immunomodulator profiling of MPE patients with the most frequent pleural effusion malignancies. This included lung adenocarcinoma, breast, and ovarian carcinoma, malignant pleural mesothelioma, and gastrointestinal carcinoma using cytologic cell blocks prepared from centrifuged pleural liquid sediment. We used immunohistochemistry (IHC) and a digitalized image analysis approach to quantify specific types of immune cells in relation to the total CD45+ leukocytes content. Subsequently, we evaluated the NanoString-derived mRNA expression levels of clinically targetable co-stimulatory and co-inhibitory genes together with the most relevant immune cells-specific marker genes. We hypothesized that immune cell ratios might be associated with patient’s clinical outcome independently of MPE tumor types, thus becoming prognostic parameters.

## 2. Results

### 2.1. Cohort Description

**Figure 1:**
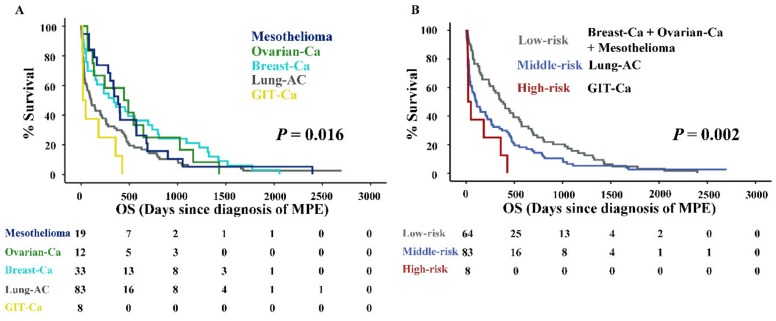
Overall survival of malignant pleural effusion (MPE) patients. (**A**) Kaplan–Meier curves of MPE patients according to the five tumor entities. (**B**) Kaplan–Meier curves of MPE patients according to the three risk categories. *p*-value of log rank test is indicated. Abbreviations: Overall survival (OS).

Within the total 155 MPE patient cohort (mean age 69 (29–93) years), the estimated median overall survival (medOS) from the diagnosis of effusion was 201 (110–291) days. According to the palliative management principle, treatments such as thoracentesis, indwelling pleural catheter, and pleurodesis were primarily performed to relieve the symptoms caused by MPE regardless of the tumor entity. Antitumor therapies were only optionally applied to patients with good systemic therapy responsiveness and functional status. A significant spread of survival times was observed among the five tumor types (Table 1). Based on this data, we grouped them into three subcategories according to their prognosis: Low-risk (Breast-Ca, Ovarian-Ca, and Mesothelioma), middle-risk (Lung-AC), and high-risk (GIT-Ca). The Kaplan–Meier survival analysis showed that both stratifications by the five tumor types (Figure 1A) as well as the three risk category groups (Figure 1B) were statistically significant (log-rank *p*-value < 0.05). The medOS from high to low-risk categories was 21, 107, and 362 days, respectively.

### 2.2. High CD20+/CD45+, but Low MPO+/CD45+ Cells Ratios by Computerized IHC Analysis, Are Generally Favourable MPE Prognosticators

**Figure 2:**
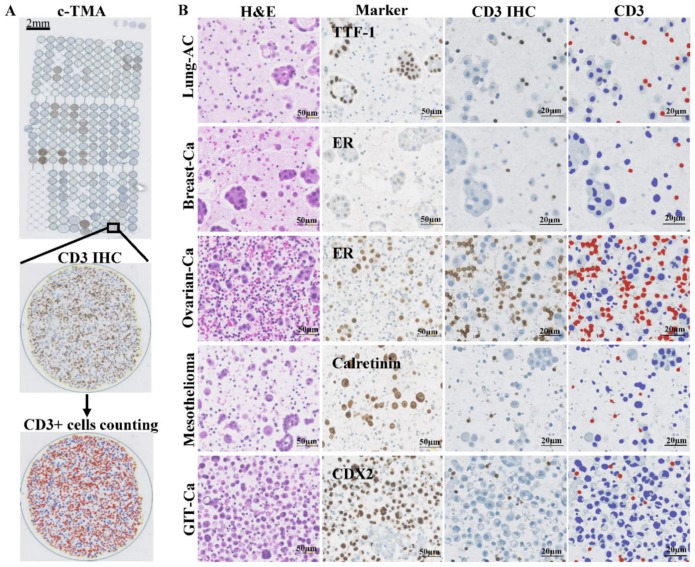
Hematoxylin-eosin (H&E) and diagnostic marker staining as well as digitalized image analysis by QuPath. (**A**) the depiction of c-TMA stained with anti-CD3 antibody followed by automated annotation of CD3+ lymphocytes. (**B**) representative images of H&E (column 1) and diagnostic marker stainings (column 2) as well as CD3 immunohistochemistry (IHC) (column 3) followed by QuPath annotation (column 4) for each tumor type. Cells with brown anti-CD3 immunoreactivity were masked red and their numbers counted per mm^2^. Unstained cells were labelled blue. Scale bar = 50 or 20 µm, respectively.

We adapted the QuPath computer algorithm for counting immune cells stained by IHC on c-TMA cores, as demonstrated (e.g., for CD3, Figure 2A,B).

**Figure 3:**
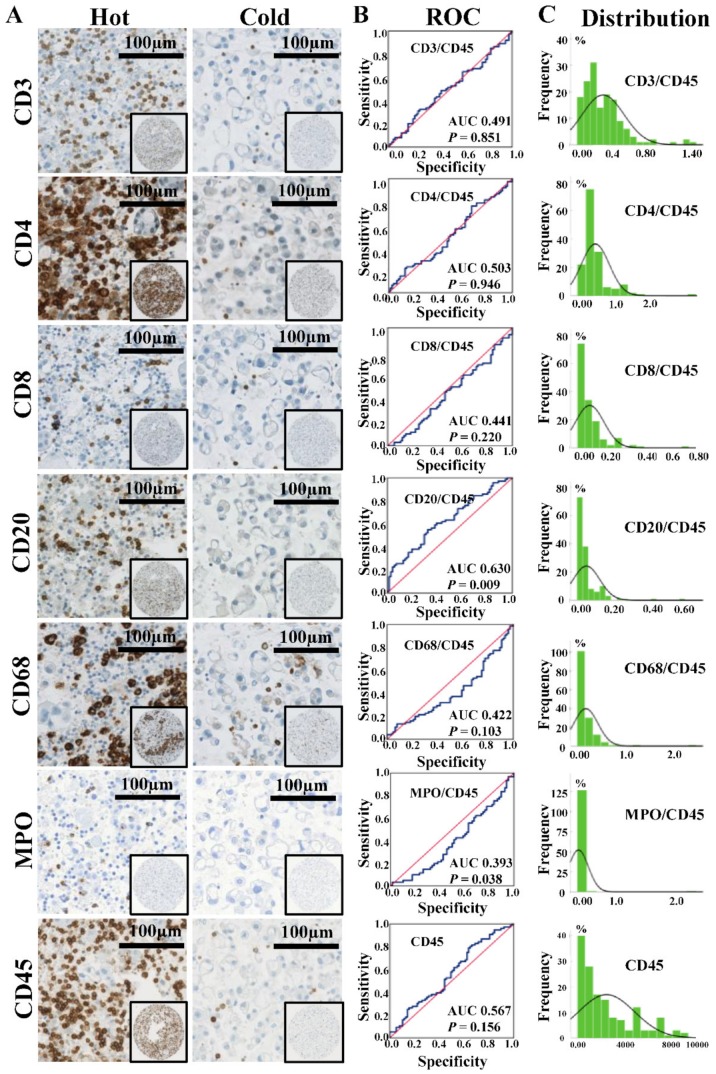
Expression of each immune cells-specific markers and corresponding receiver operating characteristic (ROC) curves analysis. (**A**) IHC stainings of immune cells-specific markers, scale bar = 100 µm. “Hot” and “cold” statuses of immune cell infiltration in MPE microenvironment were found among patients. (**B**) ROC curves analysis of each immune cell type for whole MPE patients. The cut-off value was six months (median overall survival for all MPE patients). (**C**) Distribution histograms of ratios of immune cells-specific markers to CD45+ leukocytes. The horizontal axis reflects immune cell ratio or cell counting (only for CD45, number/mm^2^), and the vertical axis reflects the number of samples. Abbreviations: Area under the curve (AUC).

With regard to the density of immune cells around cancer cells on c-TMA cores, we noted a considerable variation from low density (“cold”) to high density (“hot”) for CD3, CD4, CD8, CD20, CD68, and MPO positive cells (Figure 3A). Since CD45 is a pan-leukocyte marker, we first evaluated its prognostic potential. However, no significant survival difference was found between patients with either “cold” or “hot” MPE (median, HR = 0.96, *p*-value = 0.787). Subsequently, we analyzed CD45 normalized lineage markers, using receiver operating characteristic (ROC) curve analysis (cut off = six months, the median OS of MPE patients). As shown in Figure 3B, a high ratio of CD20+ B cells to CD45+ leukocytes (BLR) was found to be a general favorable prognosticator (area under the curve (AUC) = 0.630, *p*-value = 0.009). In contrast, a high ratio of MPO+ neutrophils to CD45+ leukocytes (NLR) was dismal (AUC = 0.393, *p*-value = 0.038). Other immune cells ratios did not reach significance (*p*-value ≥ 0.05). The ratios of immune cells-specific markers to CD45 had a nonparametric distribution (Figure 3C), therefore we further analyzed the prognostic potential of BLR and NLR using median expressions (BLR = 3.34% of CD45+ cells; NLR = 1.90% of CD45+ cells). 

**Figure 4:**
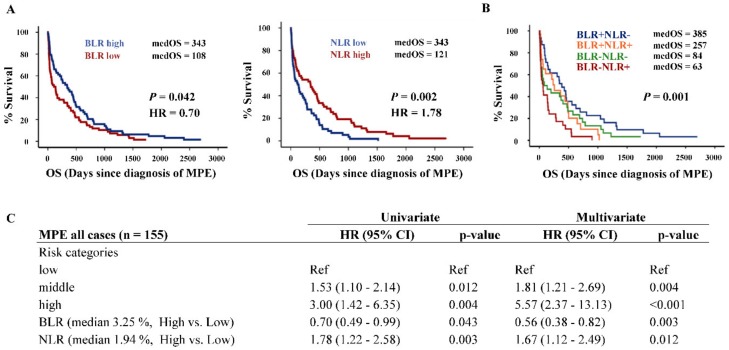
Survival analysis of BLR/NLR and risk categories. (**A**) Kaplan–Meier survival analysis of MPE patients for BLR and NLR. The curves were separated by their median expressions and correspondingly defined as “high” and “low.” (**B**) Survival analysis of combined BLR/NLR data. (**C**) Univariate and multivariate Cox regression analysis for OS. Risk categories: low (Breast-Ca, Ovarian-Ca, and Mesothelioma), middle (Lung-AC) and high (GIT-Ca). Abbreviations: Hazard ratio (HR), B cells to leukocytes ratio (BLR), neutrophils to leukocytes ratio (NLR).

Kaplan–Meier survival analysis showed that high NLR and low BLR, respectively, were poor prognostic factors for MPE patients (Figure 4A). After combining BLR and NLR expression data, we found that high BLR and low NLR expression (BLR+ NLR-) group had the highest medOS (385 days), whereas low BLR and high NLR expression (BLR- NLR+) group had the lowest medOS of 63 days (Figure 4B). Together with risk categories, NLR and BLR were generally prognostic parameters for MPE patients by both univariate and multivariate Cox regression analysis (Figure 4C). The Kaplan–Meier survival analysis was also performed for the cohort without the high-risk group (MPE GIT-Ca) in order to clarify that the prognostic significance of BLR and NLR was not influenced by the high-risk group’s extremely short survival time and small patient number. As shown in Supplementary Figure S1A,B, without the high-risk group, the BLR and NLR still kept the same prognostic potential (*p*-value < 0.05). Moreover, for only MPE Lung-AC cases (the most frequent entity causing MPE), after combining BLR and NLR expression data (Supplementary Figure S1C), the same prognostic trend was found: The BLR- NLR+ group presented significantly lower medOS of only 29 days compared to other groups together (medOS = 111 days, *p*-value = 0.018, Supplementary Figure S1D).

### 2.3. Immune Gene Signature of High B Cells and Low Neutrophils Corresponding Gene Expressions Is a Good Prognostic Parameter for MPE Patients 

We corroborated the IHC data obtained from computerized image analysis by corresponding mRNA expression analysis. Nine lineage-specific and 17 immunomodulatory genes were investigated (Supplementary Table S1). After normalization by four housekeeping genes, we observed again a significant variation of gene expression. Generally, B cells, neutrophils, T cells, macrophages, and pan-leukocytes specific gene expressions significantly correlated with their IHC quantification results with coefficients between 0.33 to 0.69 (Supplementary Figure S2). In order to validate the prognostic roles of BLR and NLR at the transcriptional level, we again normalized *CD20* (B cells specific gene) and *S100A12* (neutrophils specific gene) against *PTPRC* (CD45). Consistent with the NLR prognostic result, patients with high a *S100A12/CD45* ratio had a significantly shorter medOS (*p*-value = 0.035, Supplementary Figure S3A). Even though the survival analysis of *CD20/CD45* did not reach significance, we still observed the same survival trend of BLR that patients with high *CD20/CD45* ratio had a longer medOS (385 days versus 236 days, Supplementary Figure S3B). 

**Figure 5:**
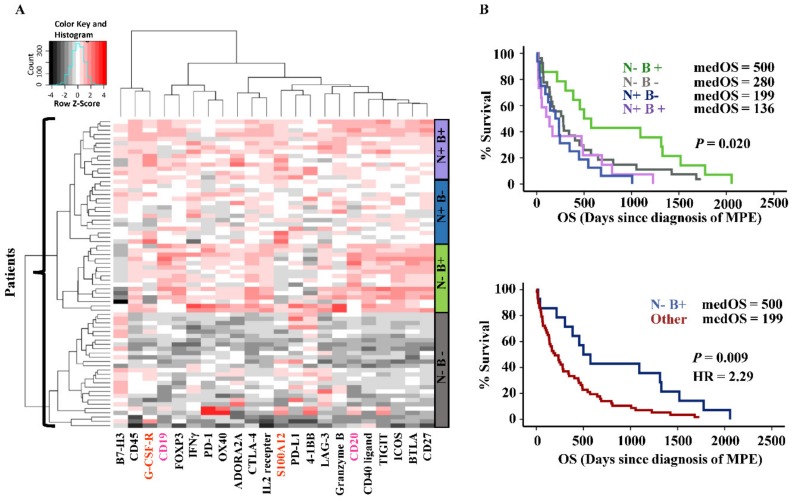
Immune gene signatures and survival analysis. (**A**) heatmap showing clusters of patients and corresponding immune gene signatures (right sidebars). Neutrophils specific gene expressions high/low is indicated as “N+/-”, and B cells corresponding gene expressions high/low is indicated as “B+/-”. Genes with high-level expressions are in red, and low-level are in black. Gene names of neutrophils and B cells lineage markers are highlighted in orange and pink, respectively. (**B**) Kaplan–Meier survival analysis according to the combinations of N+/- and B+/- and further dichotomization into N-/B+ versus the rest.

Furthermore, we performed unsupervised hierarchical clustering based on 21 genes including B cells (*CD19* and *CD20*) and neutrophils-specific genes (*S100A12* and *G-CSF-R*) together with the 17 immunomodulatory genes. Four immune gene signatures (clusters) were identified according to the expression levels of B cells and neutrophils genes (Figure 5A). In line with the survival analysis result of combined BLR/NLR data, patients with high B cells (B+) and low neutrophils (N-) gene signature had the longest medOS of 500 days (compared with the rest of the patients: *p*-value = 0.009, HR = 2.29, Figure 5B), and the corresponding immune gene signature was also independent of risk categories (tumor entities) according to multivariate Cox regression analysis (*p*-value of immune gene signature was < 0.05).

Taken together, our transcriptional result validated the prognostic roles of B cells and neutrophils for MPE patients.

### 2.4. Correlation of Immune Cell Lineage Genes with Immunomodulatory Genes

**Figure 6:**
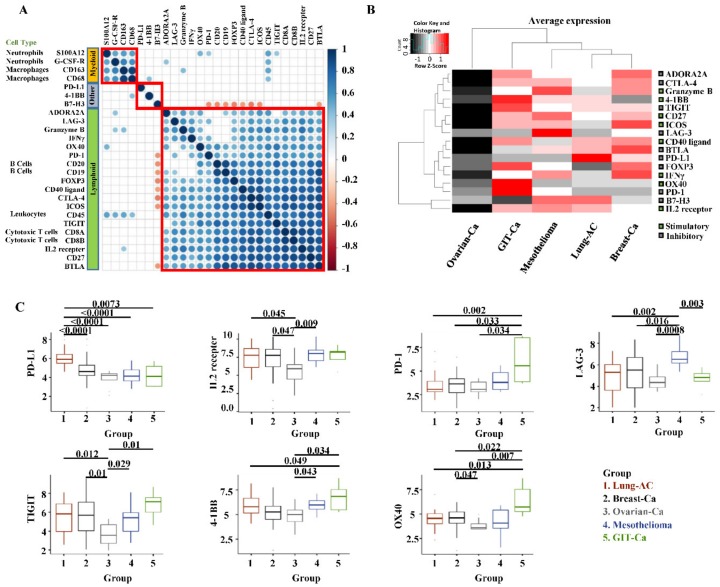
Immune cell markers and immunomodulators with correlation matrix and distribution among tumor entities. (**A**) Pearson–correlation matrix showing the clustering and correlations of 17 immunomodulators and nine immune cells-specific genes. The correlation matrix was subjected to unsupervised hierarchical clustering (Euclidean distance measurement). Blue dots illustrate positive correlation, white no correlation (FDR ≥ 0.01), and red dots illustrate negative correlation. Bigger dot size means a smaller *p*-value of the correlation. Red squares indicate the clusters. (**B**) Heatmap indicating the average mRNA expression of 17 stimulatory or inhibitory genes among five MPE tumor entities. Data were normalized and unsupervised clustered (Euclidean distance measurement). High-level expressions are in red, and low-level expressions are in black. On the right side, green squares are stimulatory genes and black squares are inhibitory genes. (**C**) mRNA expression of immunomodulators among MPE tumor entities. Markers with no statistical difference among groups (*p*-value ≥ 0.05) were not shown. *p*-value≥ 0.05 was not presented for the listed gene expression comparison between groups.

Correlation analysis may allow for better understanding of synergistic or antagonistic effects of immune cells as well as immunomodulatory molecules. As shown in Figure 6A, immune cells lineage markers of the same cell type were clustered closely with strong correlations (e.g., *CD19* and *CD20* for B cells). The correlation matrix displayed mainly three groups: 1) the myeloid group defined by lineage genes of macrophages and neutrophils, 2) the lymphoid group containing cytotoxic T cells and B cells genes together with most immunomodulatory genes, 3) the last group (named “other” in Figure 6A) including *PD-L1*, *4-1BB*, and *B7-H3*. Genes in the lymphoid group barely correlated with genes in the myeloid group, which was in line with the opposed prognostic tendency of BLR (favorable, lymphoid) and NLR (unfavorable, myeloid). *PD-L1* was clustered in an independent group showing no correlation with genes in the other two groups, suggesting that its activation might undergo a distinct pathway. Furthermore, the *CD45* gene positively correlated with all immune cell lineage genes, which proved its function as a pan-leukocyte marker for normalization.

### 2.5. Immunomodulatory Markers Expressed Differently Among MPE Tumour Entities

As for the mRNA expression of immunomodulatory genes among tumor entities (Figure 6B,C), the immune inhibitory marker *PD-L1* was particularly expressed in the MPE Lung-AC group, which was consistent with our IHC results showing that most of the PD-L1 high expression cases (17 out of 19) were found therein (Supplementary Table S2), while in MPE mesothelioma cases, *LAG-3* was dominantly expressed. For MPE Breast-Ca, *ICOS* was highly expressed. MPE GIT-Ca patients showed high expression levels of *OX40*, *4-1BB*, and *PD-1*. The Ovarian-Ca group presented lower expression levels of *TIGIT* and *IL2RB* (IL2 receptor).

## 3. Discussion

In our study, we investigated the immune MPE liquid microenvironment on aspects of immune cell type composition and expression of associated immunomodulators at the cellular and transcriptional levels using digitalized cytopathologic methods. Quantification of specific immune cell types by QuPath indicated that high BLR but low NLR were independent favorable prognosticators. This was further corroborated by transcriptional analysis of digitalized NanoString mRNA expression showing that a signature of high B cells and low neutrophil gene expressions correlated with better survival outcomes of patients.

Patients bearing MPE have a short overall survival since tumor cell entry into the pleural cavity defines advanced stage disease. Still, it was recently shown that there is considerable variation of survival time depending on tumor type, ranging from 100 to 400 days for the 50% cumulative rate [2]. This may be translated into personalized treatment regimens such as intracavitary, eventually hyperthermic, chemo/immunotherapy. Consistent with the finding by Clive et al. [2], MPE Lung-AC patients had shorter survival times compared with Breast/Ovarian-Ca and mesothelioma in our cohort. However, there was no significant difference between mesothelioma versus Breast/Ovarian-Ca patients. Rather, we identified a high-risk group of MPE patients suffering from GIT-Ca. We thus subcategorized the five tumor entities into three risk categories (low, middle, and high). The sizable survival difference between risk categories may help managing decision for individual patients.

In addition to histopathological parameters, other factors such as peripheral blood immune cell composition also correlated survival of MPE patients (e.g., “N”, the serum neutrophils to lymphocytes ratio as part of the LENT scoring system) [2,11]. Furthermore, in the microenvironment of solid tumors, the immune score—which is based on the immune cell type, density, and location of where lymphocytes infiltrate—is a novel prognostic factor [12,13]. Therefore, we hypothesized that immune cells in a liquid tumor microenvironment may also exert prognostic function, in particular due to direct contact unhindered by stromal desmoplastic fibroblasts and collagen fibers, respectively [14]. Herein, the proposed digitalized pathologic approach enables an accurate quantification of immune cells stained by IHC in relation to the amount of cancer cells per given cell block surface in mm^2^. Our method may expand the use of cytologic cell blocks for investigation of individual cell compositions. In addition, it allows automated high-throughput analysis of tissue microarray staining, and warrants each representative core being processed by the same unbiased algorithm. A drawback of the cell block technology is the variance of cellular sediment input depending on liquid volume. This is possibly the reason why a first prognostic analysis of “hot” versus “cold” effusion was not significant. One limitation of our digital image quantification algorism (QuPath) is that it is difficult to distinguish membranous staining from cytoplasmic staining of tumor cells. Therefore, we did not perform digitalized image quantification for PD-L1 IHC scoring.

Regarding the cancer immune microenvironment, immune cells are a double-edged sword for tumor progression and metastasis [15], thereby differently influencing patient survival. In our study, BLR was a favorable prognosticator for MPE patients, which may be explained by its stimulatory function in the adaptive immune system as antigen presenting cells (APCs) for CD4+ and CD8+ T cells [16]. The presence of infiltrating B cells was also linked to favorable clinical outcomes in solid tumors [17,18]. By contrast and in line with our NLR prognostic finding, a high neutrophil count in effusion liquids was associated with unfavorable clinical outcomes for patients with malignant pleural or pericardial effusions [11,19]. 

We further validated the BLR and NLR prognostic influences using multiplexed digital mRNA expression analysis by NanoString. This technology is able to detect degraded mRNA from formalin-fixed paraffin-embedded (FFPE) samples (cell blocks) without an amplification step as opposed to RT-PCR [20,21,22]. Our novel custom mRNA panel includes two parts: (1) immune cell specific markers from highly concordant and co-expressed gene sets for five immune cell types [23,24], and (2) targetable immunomodulator genes selected from a immuno-oncology experts confirmed list [25]. Interestingly, concerning the gene correlation matrix, B cell genes were co-expressed with most of the immunomodulator genes with substantially high coefficients, suggesting an underlying co-activation mechanism of multiple immune checkpoints and costimulatory molecules in the lymphoid cells related immune response. High expression of B cells and corresponding immunomodulatory genes and low expression of neutrophils genes resulted in better survival outcomes for MPE patients. Moreover, it was reported that B7-H3 protein expression positively correlated with cancer severity and poor outcome in various cancer types [9,26,27,28]. We found that *B7-H3* was the only negatively correlated gene, exclusively expressed at low levels in the highest medOS (500 days) immune gene signature group (B+, N-).

Regarding potential immunotherapeutic targets, immune checkpoints as well as immune stimulatory molecules considerably affect the efficacy of cancer immunotherapy with several agonists and antagonists being evaluated in clinical oncology [29]. In our gene profiles, some of these immunomodulators were differentially expressed among tumor entities. This may allow personalized designs of local or even systemic immunotherapy. For example, PD-1/PD-L1-targeted immunotherapy may be preferentially considered for Lung-AC MPE patients, while LAG-3 inhibitory therapy may be suitable for mesothelioma patients. To enhance therapeutic outcomes, combined immunotherapy could be considered for patients with co-expression of multiple immunomodulatory molecules such as GIT-Ca in our study. In addition, the present findings may improve the efficacy of immunomodulatory therapeutic interventions as PD-L1 expression level of tumor cells is one of the most important factors to identify suitable patients for PD-1 blockade treatment [30,31]. It is also worth noting that the Ovarian-Ca group had the lowest gene expression and immune cell infiltration among the different tumor entities. This may represent a “cold” onco-immune interaction microenvironment.

## 4. Materials and Methods 

### 4.1. Patient Cohort

Cytologic cell blocks prepared from the centrifugation sediments of 155 MPE patients in the time period of 2005 to 2013 were included in this study. Only cell blocks having >20 clusters of malignant cells per whole section surface were included. The cohort consisted of the following tumor entities: lung adenocarcinoma (Lung-AC), breast carcinoma (Breast-Ca), ovarian carcinoma (Ovarian-Ca), gastrointestinal carcinoma (GIT-Ca, including pancreas, colon, stomach, and esophagus), and pleural mesothelioma. All cases were classified based on morphology, clinical data, and IHC with respective markers. Cases with unknown primary, with other tumor types, or with potential reactive pleural effusion were excluded. Of the 155 samples, 71 were further selected for NanoString mRNA analysis. 

### 4.2. Preparation of Cellblocks and Cytologic Microarray (c-TMA)

The effusion liquids were prepared and processed as previously described [32]. Briefly, after low-speed centrifugation, the upper white phase of the sediment was aspirated and transferred into a micro tube. Thrombin and plasma were added for clot formation. After formalin fixation, clots were paraffin embedded and hematoxylin-eosin (H&E) stained. From the most representative region of the donor block, two paraffin cores of 0.6 mm diameter and 3–4 mm height were taken and precisely arrayed into a new recipient paraffin block using a custom-made, semiautomatic tissue arrayer (Beecher Instruments).

### 4.3. Immunohistochemistry

Sections with a thickness of 3 μm were cut for IHC analysis. Laboratory-developed assays were tested on a multi-tissue microarray for clones E1L3N (Cell Signaling Technology, dilution 1:100). IHC using antibodies against CD3 (mature T-cells), CD4 (helper T-cells), CD8 (cytotoxic T cells, Tc cells), CD20 (B cells), CD45 (leukocytes), CD68 (macrophages), myeloperoxidase (MPO, neutrophilic granulocytes), and diagnostic markers for each MPE tumor entity. Thyroid transcription factor 1 (TTF-1), CDX2, estrogen receptor (ER) and calretinin were performed on a Benchmark Ultra platform (Ventana) with protocols used for routine diagnostics.

### 4.4. Scoring and Digitalized Image Analysis

IHC stainings were scanned by a high-resolution scanner (Nanozoomer Digital Pathology). Considering that the density of MPE leukocytes per 2D cell block core surface is dependent on the liquid volume used for centrifugation before manufacturing of cell block, the total number of immune cells is of limited value. Therefore, the expression values of other immune cells-specific markers were normalized against CD45 expression. Ratios of CD3, CD4, CD8, CD20, CD68, and MPO-positive immune cells were calculated using QuPath [33], an open-source software for quantitative pathology (Queen’s University, Belfast, Northern Ireland), as follows: (Positive cell count/mm^2^)/(CD45+ cell count/mm^2^). Briefly, after TMA de-arraying and tissue detection of each TMA core, the image was then performed by “estimate stain vectors” function to adjust the RGB values. Afterwards, a representative core with IHC positive staining was analyzed by the “positive cell detection” algorism using “optical density sum” for the image detection. Other parameters such as “sigma”, “threshold”, and ”background radius” were adjusted by staining until a decent (positive) cell detection was achieved. Then, the algorism with fixed parameters was automatedly applied to the rest of the TMA cores to quantify the tissue area, total cell number, and positive cell number for each TMA core. The prognostic contribution of each marker was subsequently assessed using ROC curve analysis (cut-off value was the medOS of MPE patients, six months). TTF-1, ER, calretinin and CDX2 IHC stainings were scored 0 (negative) or 1 (positive). PD-L1 immunoreactivity was dichotomized into low (<50%) and high (≥50%), considering only membranous but not cytoplasmic staining of tumor cells.

### 4.5. RNA Extraction and Digital Immune Gene Expression Analysis

Total RNA was extracted from the cellblock whole sections (two cut sections, each 5 µm thick) using the Maxwell purification system (Maxwell RSC RNA FFPE Kit, AS1440, Promega). RNA was eluted in 50 µl RNase-free water and stored at −80 °C. RNA concentration was measured using a Qubit 2.0 fluorometer (Life Technologies) appertaining the RNA broad-range assay. RNA integrity was assessed using a fragment analyzer (Agilent Technologies) appertaining DNF-489 standard sensitivity RNA analysis kit. After mRNA quality control, 71 MPE patient samples were randomly selected for the following analysis. Gene expression patterns were screened using the NanoString nCounter platform for digital gene expression analysis with a custom-made PlexSet panel for simultaneous measurement of up to 96 samples each single run. The panel included 17 co-stimulatory or co-inhibitory genes together with T cells, B cells, macrophages, leukocytes, and neutrophils-specific marker genes (nine in total) and four housekeeping genes for normalization. Probes were hybridized to 50 ng of total RNA input for 20 h at 65 °C and applied to the nCounter preparation station. The post-hybridization processing was performed by using the nCounter Max/Flex System using the high-sensitivity protocol and the cartridge was scanned and read on the digital analyzer at 555 FOV. mRNA expression data will be deposited in a publicly available database during review.

### 4.6. Digital Data Processing

NanoString data processing was performed with the R statistical programming environment (v3.4.2) using the NanoStringNorm and the NAPPA package. Considering the counts obtained for positive control probe sets, raw NanoString counts for each gene were subjected to a technical factorial normalization, carried out by subtracting the mean counts plus 2× standard deviation from the CodeSet inherent negative controls. Afterward, a biological normalization using a factor calculated out of the ratio between geometric mean of the included reference genes in each sample and the geometric mean of all calculated geometric means. Additionally, all counts with *p*-value ≥ 0.05 after one-sided t-test versus negative controls plus 2× standard deviations were interpreted as not expressed to overcome basal noise.

### 4.7. Statistical Analysis

All statistical analyses were performed on SPSS software, version 23 (IBM) or environment R, version 3.4.2 (R Core Team). Overall survival (OS) was defined as the period from the date of first MPE diagnosis to patients’ death, and was computed using the Kaplan–Meier method and log-rank tests. Univariate Cox regression analyses were used for prognostic marker evaluation. Significant parameters and clinically relevant parameters were introduced into multivariate Cox regressions. Receiver operating characteristic (ROC) curve was performed to assess the diagnostic contribution of immune cells markers. The Shapiro–Wilks test was applied to test for normal distribution of each data set. Based on the results, for dichotomous variables either the Wilcoxon Mann–Whitney rank sum test (non-parametric) or the two-sided Student’s t-test (parametric) was used. For ordinal variables with more than two groups, either the Kruskal–Wallis test (non-parametric) or ANOVA (parametric) was used to detect group differences. Correlation matrices were created with Pearson’s correlation. Correlations between mRNA and IHC data were tested using the Spearman’s rank correlation tests (rho). The *p*-value was adjusted for multiple comparisons by using the false discovery rate (FDR, Benjamini–Hochberg method). A *p*-value < 0.05 was considered significant.

## 5. Conclusions

Digitalized immune cell profiling together with immunomodulator transcriptional profiling demonstrated the complexity and heterogeneity of the MPE microenvironment in terms of the cellular composition and targetable mRNA expression levels. Even though external cohorts wouldbe needed to further strengthen the prognostic findings in future studies, we were still able to highlight the influence of B cells/leukocytes ratio, neutrophils/leukocytes ratio, and corresponding immune gene signatures on MPE patients. This comprehensive investigation of MPE cancer immunity offers new insight into diagnostic and prognostic cytopathology evaluation and may lead to better therapeutic strategies and management for MPE patients.

## Figures and Tables

**Table 1:** Cohort description.

Tumor Type	Lung-AC	Breast-Ca	Ovarian-Ca	Mesothelioma	GIT-Ca	Total
Frequency n (%)	83 (52)	33 (21)	12 (8)	19 (12)	8 (5)	155 (100)
Age median (range)	70 (29–93)	68 (34–87)	64 (42–81)	67 (47–85)	70 (56–82)	69 (29–93)
Sex M/F	45/38	1/32	0/12	17/2	5/3	68/87
OS median days (95% CI)	107 (44–170)	341 (92–560)	446 (28–864)	385 (293–477)	21 (0–60)	201 (110–291)
OS mean days (95% CI)	340 (223–457)	562 (363–763)	532 (273–791)	515 (275–756)	136 (19–253)	403 (328–494)
Diagnostic markers	TTF-1	ER	ER	Calretinin	CDX2	
Positive n (%)	71 (85)	28 (85)	11 (92)	19 (100)	8 (100)	137 (88)
Negative n (%)	12 (15)	5 (15)	1 (8)	0	0	18 (12)
Nanostring mRNA test n	20	27	8	11	5	71

Abbreviations: Overall survival (OS), confidence interval (CI), lung adenocarcinoma (Lung-AC), breast carcinoma (Breast-Ca), ovarian carcinoma (Ovarian-Ca), gastrointestinal carcinoma (GIT-Ca), thyroid transcription factor 1 (TTF-1), estrogen receptor (ER).

## Supplementary Materials

The following are available online at https://www.mdpi.com/2072-6694/11/12/1953/s1, Figure S1: Survival analysis of BLR and NLR. Figure S2: Immune cells-specific markers correlation analysis between computational IHC quantification and NanoString mRNA results. Figure S3: Survival analysis of *S100A12/CD45* and *CD20/CD45*. Table S1: mRNA list and category. Table S2: Digitalized image quantification results for immune cell markers and PD-L1 scoring.
