# Supplementary material for: Prognostic Immune Cell Profiling of Malignant Pleural Effusion Patients by Computerized Immunohistochemical and Transcriptional Analysis
Source: Cancers (Basel). 2019 Dec 5;11(12):1953. doi: 10.3390/cancers11121953 (PMC6966627; doi:10.3390/cancers11121953)
Supplement: Supplementary file 1 [file cancers-11-01953-s001.pdf]

cancers MDPI

Supplementary Materials: Prognostic Immune Cell Profiling of Malignant Pleural Effusion Patients by Computerized Immunohistochemical and Transcriptional Analysis

Table S1. mRNA list and category. NOTE: a, the drug list does not include all available agonists or antagonists for each target.

Gene name	Common name	Category	Subcategory	Drug[a]
CD27	CD27	Immunomodulator	Stimulatory	Varlilumab
CD40LG	CD40 ligand	Immunomodulator	Stimulatory	SEA-CD40
GZMB	Granzyme B	Immunomodulator	Stimulatory	
ICOS	CD278	Immunomodulator	Stimulatory	GSK3359609
IL2RB	IL2 recepter	Immunomodulator	Stimulatory	Aldesleukin
TNFRSF4	OX40	Immunomodulator	Stimulatory	PF-8600
TNFRSF9	4-1BB	Immunomodulator	Stimulatory	Utomilumab
ADORA2A	Adenosine $A2_A$ receptor	Immunomodulator	Inhibitory	AZD4635
BTLA	BTLA	Immunomodulator	Inhibitory	preclinical trials
CD274	PD-L1	Immunomodulator	Inhibitory	Atezolizumab; Durvalumab
CD276	B7-H3	Immunomodulator	Inhibitory	Enoblituzumab
CTLA4	CTLA-4	Immunomodulator	Inhibitory	Ipilimumab
FOXP3	FOXP3	Immunomodulator	Inhibitory	
IFNG	IFNγ	Immunomodulator	Inhibitory	Interferon gamma-1b
LAG3	LAG-3	Immunomodulator	Inhibitory	Relatlimab
PDCD1	PD-1	Immunomodulator	Inhibitory	Nivolumab; Pembrolizumab
TIGIT	TIGIT	Immunomodulator	Inhibitory	BMS-986207
CD19	CD19	Immune cell type	B cells	
MS4A1	CD20	Immune cell type	B cells	
CD8A	CD8A	Immune cell type	Cytotoxic T cells	
CD8B	CD8B	Immune cell type	Cytotoxic T cells	
PTPRC	CD45	Immune cell type	Leukocytes	
CD163	CD163	Immune cell type	Macrophages	
CD68	CD68	Immune cell type	Macrophages	
CSF3R	G-CSF-R	Immune cell type	Neutrophils	
S100A12	S100A12	Immune cell type	Neutrophils	
FCF1	FCF1	Housekeeping gene		
ERCC3	ERCC3	Housekeeping gene		
CNOT4	CNOT4	Housekeeping gene		
TLK2	TLK2	Housekeeping gene		

Table S2. Digitalised image quantification results for immune cell markers and PD-L1 scoring.

Title	Score	Lung-AC	Breast-AC	Ovarian-Ca	Mesothelioma	GIT-Ca	Total
CD3 count/mm^2, median		216	1060	139	253	300	394
CD3/CD45 % median		23.1	41.3	27.2	33.7	19.4	29
CD4 count/mm^2, median		1324	2229	1080	2180	1079	1578
CD4/CD45 % median		83.4	83.4	98.2	88.1	54.2	81
CD8 count/mm^2, median		70	181	44	89	40	85
CD8/CD45 % median		4.00	8.80	5.00	6.30	3.20	5
CD20 count/mm^2, median		36	54	17	44	64	43
CD20/CD45 % median		3.7	2	3	2.8	4	3
CD45 count/mm^2, median		2293	2353	1552	2962	1897	2212
CD68 count/mm^2, median		700	802	521	1287	1192	900
CD68/CD45 % median		45.5	37.1	56.9	74.1	41.6	51
MPO count/mm^2, median		15	11	22	7	19	15
MPO/CD45 % median		2.3	0.9	4	0.6	1.5	2
PD-L1 (E1L3N) n (%)	Positive	17 (21)	2 (6)	0	0	0	19 (13)
	Negative	64 (79)	31 (94)	12 (100)	18 (100)	8 (100)	133 (87)

Abbreviations: lung adenocarcinoma (Lung-AC), breast carcinoma (Breast-Ca), ovarian carcinoma (Ovarian-Ca), gastro-intestinal carcinoma (GIT-Ca).

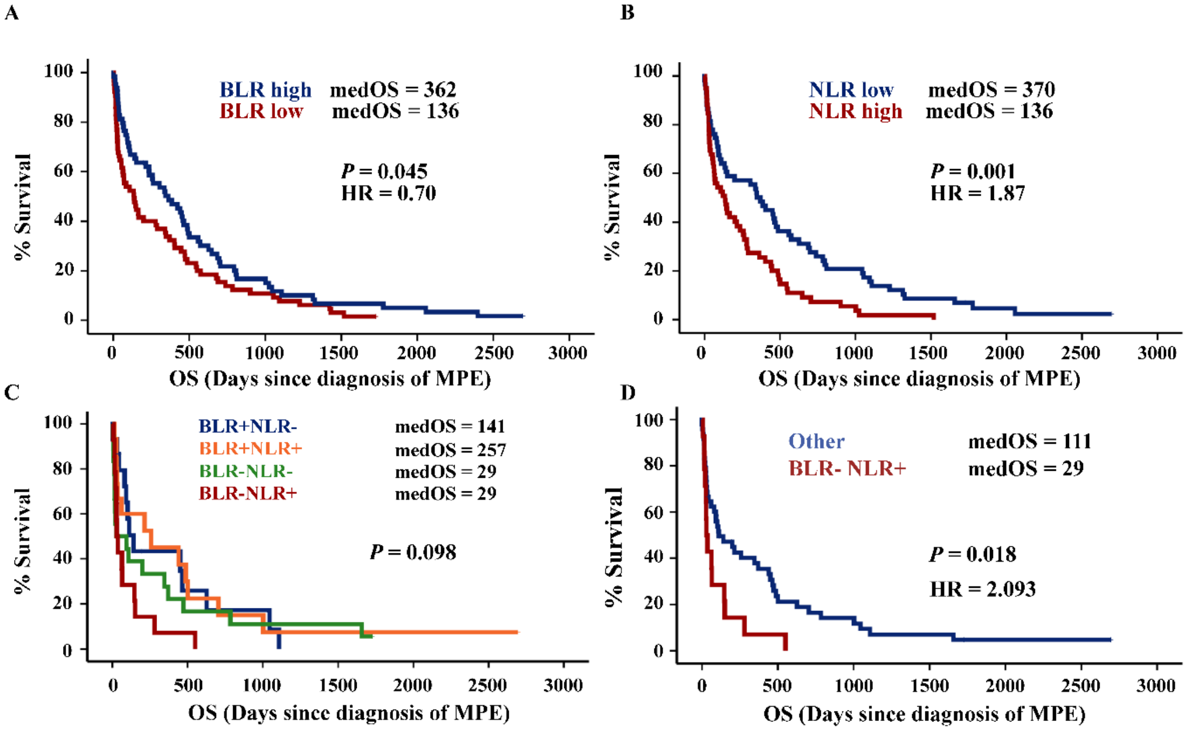

Figure S1. Survival analysis of BLR and NLR. Survival analysis of BLR (A) and NLR (B) without MPE GIT-Ca cases. Survival analysis of combined BLR/NLR data (four groups) (C) and BLR- NLR+ group vs. the rest "Other" group (D) for only MPE Lung-AC cases. The curves were separated by their median expressions, and correspondingly defined as "high" and "low". Abbreviations: Hazard ratio (HR), B cells to leukocytes ratio (BLR), neutrophils to leukocytes ratio (NLR).

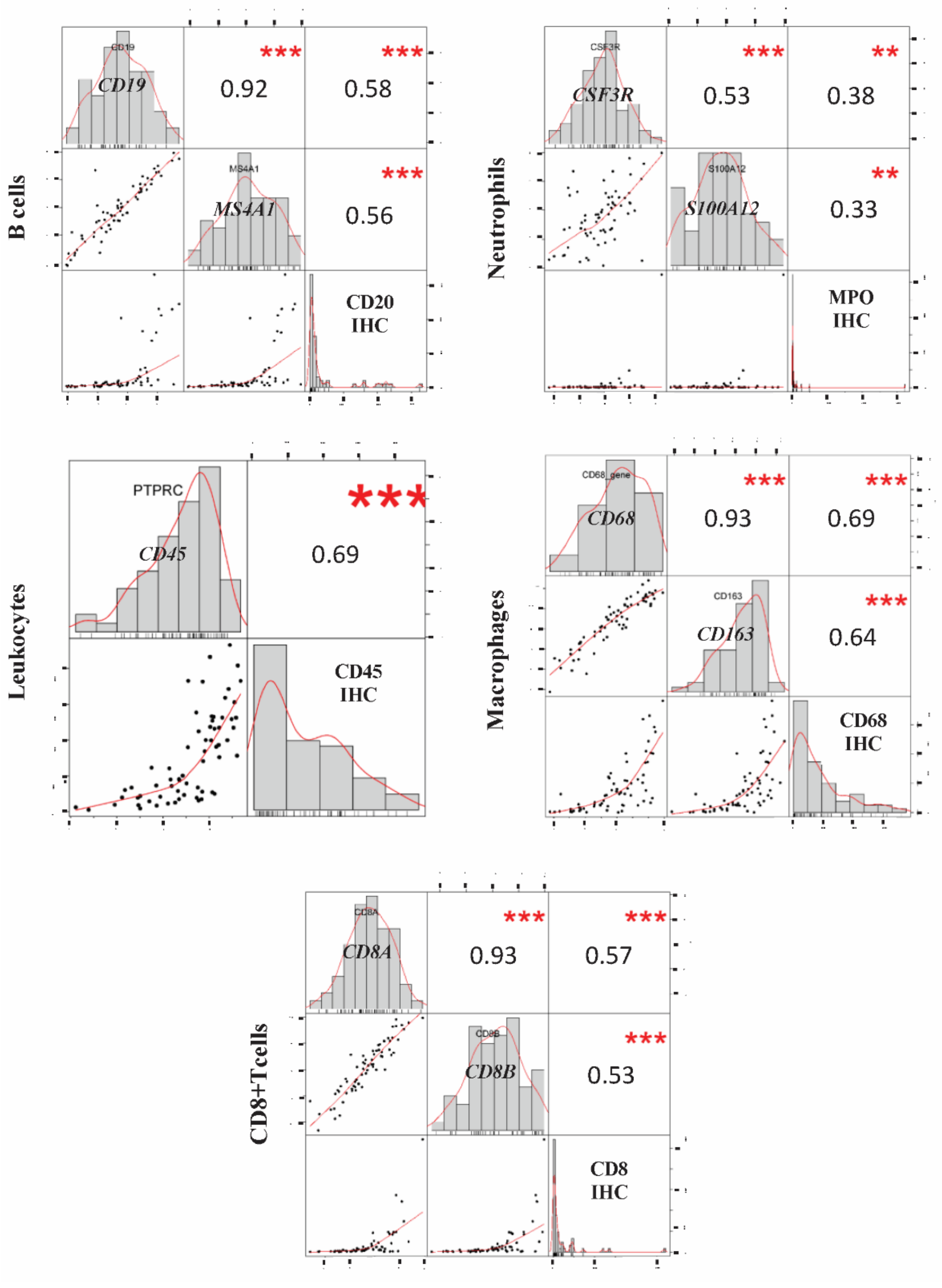

Figure S2. Immune cells-specific markers correlation analysis between computational IHC quantification and NanoString mRNA results. CD8, CD20, CD45, CD68, and MPO IHC results were correlated with corresponding mRNA expression levels. Coefficients and p-values were shown in the upper right squares. The unit of IHC quantification data is number/mm^2. P-values are presented using ** (< 0.01) or *** (< 0.001).

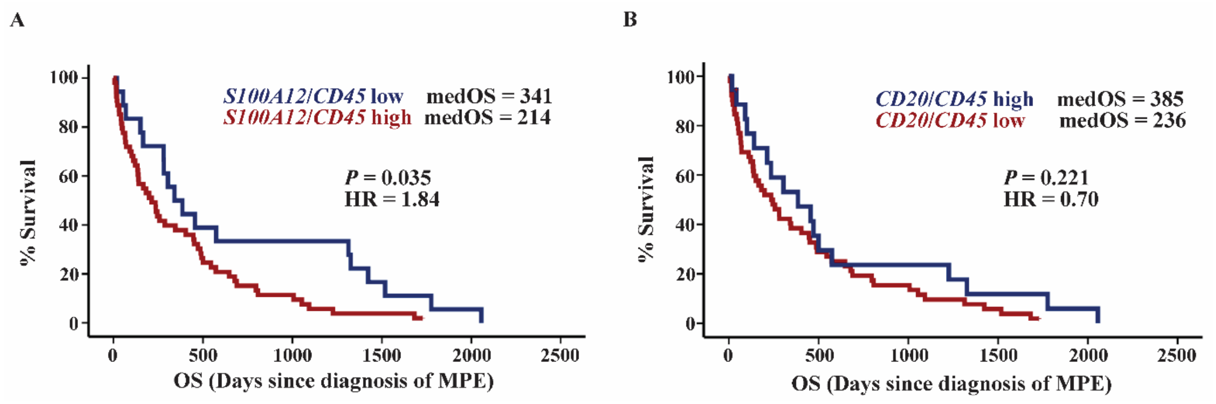

Figure S3. Survival analysis of S100A12/CD45 and CD20/CD45. (A) Kaplan-Meier survival analysis of MPE patients for S100A12/CD45. The curves were separated by the lower quartile of expressions, and correspondingly defined as "high" and "low". (B) Kaplan-Meier survival analysis of MPE patients for CD20/CD45. The curves were separated by the upper quartile of expressions, and correspondingly defined as "high" and "low". Abbreviations: Hazard ratio (HR), median overall survival (medOS).
